# The correlation between the transverse rectal diameter and urodynamic findings in children with neurogenic bowel and bladder dysfunction

**DOI:** 10.3389/fped.2022.957123

**Published:** 2022-09-29

**Authors:** Sasa Milivojevic, Aleksandra Zelenovic, Jelena Milin-Lazovic, Ognjen Radojicic, Darko Laketic, Ivana Dasic, Natasa Milic, Zoran Radojicic

**Affiliations:** ^1^Department of Urology, University Children's Hospital, Belgrade, Serbia; ^2^Institute of Anatomy “Niko Miljanic”, Faculty of Medicine, Belgrade, Serbia; ^3^Institute for Medical Statistics and Informatics, Faculty of Medicine, University of Belgrade, Belgrade, Serbia; ^4^Clinic for Gynecology and Obstetrics “Narodni Front”, Belgrade, Serbia; ^5^Department of Internal Medicine, Mayo Clinic, Rochester, MN, United States

**Keywords:** transverse rectal diameter, urodynamic findings, neurogenic bowel, bladder dysfunction, spina bifida

## Abstract

**Background:**

The aim of this study was to examine the correlation between the transverse rectal diameter and urodynamic findings in children with neurogenic bowel and bladder dysfunction.

**Methods:**

Between 2014 and 2022, we prospectively evaluated 81 consecutive spina bifida children with neurogenic bowel and bladder dysfunction (35 boys and 46 girls, mean age 9.5 ± 3.4 years). All patients underwent echosonographic measurement of transverse rectal diameter and urodynamic studies.

**Results:**

We found a strong negative correlation between transverse rectal diameter and maximum bladder capacity (*r* = −0.682, *p* < 0.001) and compliance (*r* = −0.690, *p* < 0.001). There was also a strong positive correlation between transverse rectal diameter and maximal detrusor pressure (*r* = 0.650, *p* < 0.001), leak point pressure (*r* = 0.793, *p* < 0.001), and PVR (*r* = 0.762, *p* < 0.001). In ROC analysis, transverse rectal diameter demonstrated good performance for distinguishing children with upper urinary tract deterioration, with an AUC of 0.857 (95% CI 0.761–0.953). A transverse rectal diameter ≥40 mm was 83.3% sensitive and 100% specific for the diagnosis of unfavorable urodynamic patterns.

**Conclusion:**

There is a correlation between the transverse rectal diameter and urodynamic findings in children with neurogenic bowel and bladder dysfunction. Ultrasonographically assessed transverse rectal diameter of ≥40 mm may be used as a risk factor for upper urinary tract deterioration (unfavorable urodynamic findings). We suggest the transverse rectal diameter echosonographic measurement use as an integral part of the diagnostic approach in children with neurogenic bowel and bladder dysfunction, as it can help decision-making while waiting for urodynamic testing.

## Introduction

A large amount of neurogenic bladder and bowel dysfunction cases in the pediatric population is accounted for by congenital dysraphism. Less frequent etiologies consist of anorectal or urological malformations such as the imperforate anus or bladder exstrophy, in addition to damage obtained from brain trauma, central nervous system tumors, or spinal cord injuries. Moreover, disturbances regarding the urinary sphincter muscles, sensory and/or motor input to the bladder, and anal sphincter have been observed to result in gastrointestinal and urinary dysfunction (1).

Typically, children who suffer from neurogenic bladder dysfunction originating as a result of spina bifida possess detrusor overactivity (DO) and detrusor sphincter dyssynergia (DSD), along with overactivity in the pelvic floor musculature, incontinence, an unmanageable and excessively active bladder, and functional bladder outlet obstruction. Similarly, in most cases, the spasm of the pelvic floor and the external anal sphincter are responsible for an elevated degree of constipation, which is frequently linked with incontinence (2).

For a long time, it has been generally acknowledged that constipation is capable of affecting lower urinary tract dysfunction in children who do not have other urological deviations (3, 4).

Conversely, the literature that is currently accessible highlights a limited number of papers studying the connection with respect to bowel and bladder function in patients suffering from neurogenic bladder and bowel dysfunction, with varying outcomes being observed.

An alternative method that is considered to be both reliable and noninvasive for the assessment of the rectal filling state is the echosonographic measurement of transverse diameter. This method has the possibility of overruling digital rectal observations utilized in the course of investigation with regard to children who have constipation (5–7).

This study examines the correlation between the transverse rectal diameter and urodynamic findings in children with neurogenic bowel and bladder dysfunction.

## Materials and methods

A study was organized in the urology department at the University Children's Hospital in Belgrade between 2014 and 2022, with the primary aim of investigating children suffering from neurogenic bladder and bowel dysfunction.

In that interval, a total of 81 subsequent children suffering from spinal dysraphism whose parents gave their consent to participate in the study and who sufficiently met the mandatory requirements were carefully supervised, thus sequentially being included in the study.

Certain qualifications were necessary to be fulfilled by the patients at the time of the recruitment process between June 2014 and January 2022 to be engaged in the study. Only the subjects with detrusor sphincter dyssynergia (DSD) and detrusor overactivity (DO) were admitted into the study, confirming the grounds of cytometry (urodynamic testing).

All of the patients were on the clean intermittent catheterization (CIC) and anticholinergic therapy regimen during and before the study, while 42 patients were also on the bowel management regimen in addition to the aforementioned therapy.

Throughout the study, the conditions regarding the practice of clean intermittent catheterization (CIC) and utilization of anticholinergics remained the same. Moreover, no intercurrent operative corrections to the spine or bladder had occurred.

A prescription of anticholinergic therapy including oxybutynin 0.2 mg/kg/dose was prescribed for each patient three times per day with adaptation according to their body weight.

Moreover, each patient was frequently provided with CIC *via* either native urethra or a continent vesicostomy, every 3 h.

Furthermore, each participant was recommended laxative application, daily enema, and a unique diet as part of the bowel management therapy. In contrast, a number of participants declined bowel management, which had been offered due to the parents and children lacking motivation in administrating bowel management in private surroundings as a result of the complex protocol, even though the instructions and benefits were clearly explained.

In brief, bowel management included a diet regimen, retrograde transanal enemas of the order 10–20 ml/kg/day, a maximum of 1 L of physiological solution with an addition of glycerine, and if necessary, digital rectal stimulation and suppositories (bisacodyl sup 10 mg). In addition, all the patients were prescribed polyethylene glycol laxatives with an additional 0.7 g/kg/day of electrolytes. As two patients had to undergo surgery, a Malone continent appendicectomy was drawn through the front abdominal wall at the belly button, through which daily antegrade enema was administrated.

Each patient took part in an echosonographic measurement of the transverse rectal diameter and urodynamic studies, which were later compared as part of the determination of the treatment results, for the duration mentioned above.

In brief, the **urodynamic investigation** was conducted according to the International Children's Continence Society (ICCS) standards (8). Vesical pressure was recorded with a 6 Fr. double lumen transurethral catheter, and abdominal pressure with an 8 Fr. catheter in a small rectal balloon. The activity of the striated pelvic floor muscles was monitored by skin electrodes on the perineum. The bladder was filled with saline warmed to body temperature at a rate of 5–20 ml/min and adjusted to a maximum of 10% of expected bladder capacity for an age per minute. Bladder compliance was calculated during the filling of the bladder at two-thirds of maximum bladder capacity by dividing volume by milliliters and pressure in centimeters of water. Maximal cystometric bladder capacity was defined by the maximum volume instilled at 40 cm water pressure or by the volume instilled before the start of urinary leakage. Detrusor overactivity was diagnosed when repeatedly contractions over 15 cm water pressure are seen. Detrusor leak point pressure (LPP) was considered the least detrusor pressure value in which urine loss occurred in the absence of detrusor contraction or increase in abdominal pressure. The peak detrusor pressure was calculated as the maximal pressure recorded by the cystometric curve during the filling phase in a calm child. Immediately following voiding, post-void residual volume (PVR) was recorded by catheterization in children who could void volitionally. Bladder filling was interrupted when the patient presented with bladder discomfort, strong voiding desire, continuous urinary leak, or when the pressure reached 40 cmH_2_O. We also calculated age-adjusted maximal cystometric bladder capacity.

Enclosed within this investigation, the risk factors associated with urodynamics with regard to upper urinary tract deterioration (undesirable urodynamic results) have been described as maximum detrusor pressure of 40 cm H_2_O or larger at the time of leak or filling, as well as bladder compliance being recorded as below 10 ml/cm H_2_O.

### Transverse rectum ultrasound

During the cystometry *via* ultrasonography, the measurements and recordings regarding the transversal rectal diameter were obtained. The ultrasound was performed with the patient lying face upwards as explained by Klijn et al. (9). A 7.5 MHz probe was implemented roughly 2 cm above the symphysis on the abdominal skin. The results were recorded using a reasonably full bladder capacity spanning between 30 and 70% with regard to age, at an angle of ~15 degrees below the transverse plane. Two measurements were taken with respect to the diameter of the rectum posterior of the bladder, with an average between the two recordings being determined for each patient. Each patient was questioned in order to determine if they experienced any sort of urge for excretion while the investigation was taking place and the amount of time in which the patients had defecated before the investigation. If the patient had undergone excretion within the previous 2 h or had experienced an urge to do so throughout the investigation, the procedure would be postponed until the necessary conditions related to intestinal emptying are met.

### Statistical analysis

Descriptive statistics were calculated for the baseline demographic and clinical features as well as treatment outcomes. Continuous variables were presented as means with standard deviations or medians with 25–75 percentile according to the data distribution. Categorical variables are presented with numbers and percentages. Maximal bladder capacity values were adjusted for age according to the formula 30 + (30 × age in years) for age up to 12 years, while after 12 age-expected bladder capacity is maintained at 400 ml. The correlation between BWT and urodynamic parameters was assessed by Spearman's correlation coefficient. The ROC curve was used to visualize the association between the urodynamically proved unfavorable prognosis and transversal rectal diameter. The area under the curve (AUC) was also estimated. Sensitivity, specificity, and positive and negative predictive values are calculated with corresponding 95% confidence intervals. Urodynamic parameters are compared according to transversal rectal diameter and presented as median (25–75 percentile) and analyzed with the Mann-Whitney test. A *p*-value of <0.05 was considered statistically significant. Statistical analysis was performed by IBM SPSS Statistics 21 (IBM SPSS Inc., Chicago, IL).

## Results

Baseline clinical characteristics of children are presented in Table 1. Children were 9.5±3.4 years old, with 35 (43.2%) boys and 46 (56.8%) girls.

**Table 1:** Baseline demographic and clinical features of study population according to tranversal rectal diameter.

**Variable**	***n*** **= 81**	**<40 mm**	**>40 mm**	***p***
Age, yrs, mean ± sd	9.5 ± 3.4	9.8 ± 3.4	9.1 ± 3.3	
**Gender**, ***n*** **(%)**				
Boys	35 (43.2)	19 (46.3)	16 (40)	0.565
Girls	46 (56.8)	22 (53.7)	24 (60)	
**Spina bifida**, ***n*** **(%)**				
Aperta	63 (77.8)	31 (75.6)	32 (80)	0.635
Occulta	18 (22.2)	10 (24.4)	8 (20)	
**Level of lesion**, ***n*** **(%)**				
L5 or above	58 (71.6)	30 (73.2)	28 (70)	0.752
S1 or bellow	23 (28.4)	11 (26.8)	12 (30)	
**Type of spina bifida**, ***n*** **(%)**				
Myelomeningocele	45 (55.6)	24 (58.5)	21 (52.5)	0.637
Meningocele	9 (11.1)	5 (12.2)	4 (10)	
Lipoma	15 (18.5)	8 (19.5)	7 (17.5)	
Indefinite type	12 (14.8)	4 (9.8)	8 (20)	
Associated hydrocephalus, *n* (%)	45 (55.6)	21 (51.2)	24 (60)	0.427
VCUG done, *n* (%)	32 (39.5)	15 (36.6)	17 (42.5)	–
**VUR confirmed**, ***n*** **(%)**				
Low-grade VUR^*^	6 (7.4)	5 (12.2)	1 (2.5)	0.586
High-grade VUR^**^	26 (32.1)	10 (24.4)	16 (40)	
Antibiotic prophylaxis^¥^, *n* (%)	18 (22.2)	11 (26.8)	7 (17.5)	
Continent vesicostomy done, *n* (%)	42 (51.9)	17 (41.5)	25 (62.5)	0.058
Uretherovesicostomy^y^	26 (32.1)	11 (26.8)	15 (37.5)	0.174
Appendicovesicostomy	14 (17.3)	6 (14.6)	8 (20)	
prepucial vesicostomy	2 (2.5)	0 (0)	2 (5)	
**Treatment administered earlier**				
Oxybutynin + CIC	39 (48.1)	0 (0)	39 (97.5)	< 0.001
Oxybutynin + CIC+ Bowel management^π^	42 (51.9)	41 (100)	1 (2.5)	
Orthopedic surgical intervention on hip, knee and foot deformities, *n* (%)	20 (24.7)	10 (24.4)	10 (25)	0.949
Bed-ridden patients, *n* (%)	20 (24.7)	8 (19.5)	12 (30)	0.274

VCUG, voiding cystourethrograms; VUR, vesicoureteral reflux.

* I, II and III degree, endoscopically treated with Deflux paste.

** IV and V degree, surgically treated with execution of ureteroneocystostomy.

y From distal end of ureter after executed ureteroneocystostomy.

¥ Administered during the observation period (one-third of therapy dose every evening before bed).

π Bowel management quit at least 12 months before the study began.

NA, not applicable.

Spina bifida aperta was present in 63 (77.8%), while spina bifida occulta was present in 18 (22.2%) children. A total of 58 children had lesion L5 or above, while 23 had lesion S1 or below.

In the study population, we found a strong negative correlation between transverse rectal diameter and maximum bladder capacity (*r* = −0.682, *p* < 0.001) and compliance (*r* = −0.690, *p* < 0.001).

There was a moderate positive correlation between transverse rectal diameter and maximal detrusor pressure (*r* = 0.650, *p* < 0.001). In addition, there was a strong positive correlation between transverse rectal diameter and leak point pressure (*r* = 0.793, *p* < 0.001) and PVR in our patients who could achieve spontaneous voiding (*r* = 0.762, *p* < 0.001).

When maximal bladder capacity was expressed as % EBC adjusted for age, there was a moderate negative correlation with the transverse rectal diameter (*r* = −0.474, *p* < 0.001).

In addition, when PVR was expressed as % EBC adjusted for age, there was a strong positive correlation with the transverse rectal diameter (*r* = 0.648, *p* < 0.001).

**Figure 1:**
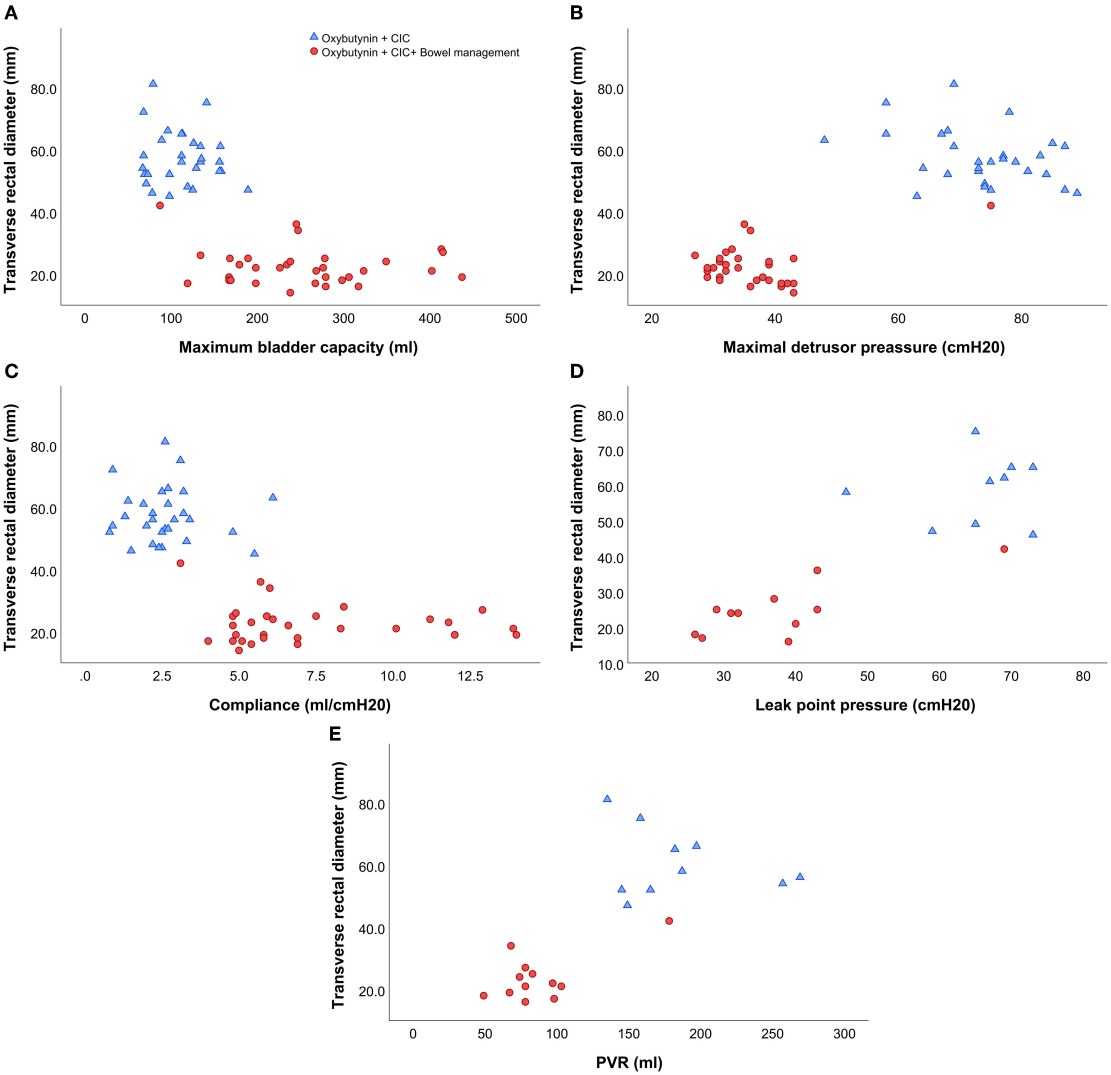
The correlation between urodynamic parameters and transverse rectal diameter stratified by therapy regimen. **(A)** The correlation between transverse rectal diameter and maximum bladder capacity. **(B)** The correlation between transverse rectal diameter and maximal detrusor pressure. **(C)** The correlation between transverse rectal diameter and compliance. **(D)** The correlation between transverse rectal diameter and leak point pressure. **(E)** The correlation between transverse rectal diameter and PVR.

The correlation between urodynamic parameters and transverse rectal diameter stratified by therapy regimen (oxybutynin + CIC vs. oxybutynin + CIC + Bowel management) is presented in Figure 1. Children with bowel management had a smaller transverse rectal diameter and favorable outcomes.

**Figure 2:**
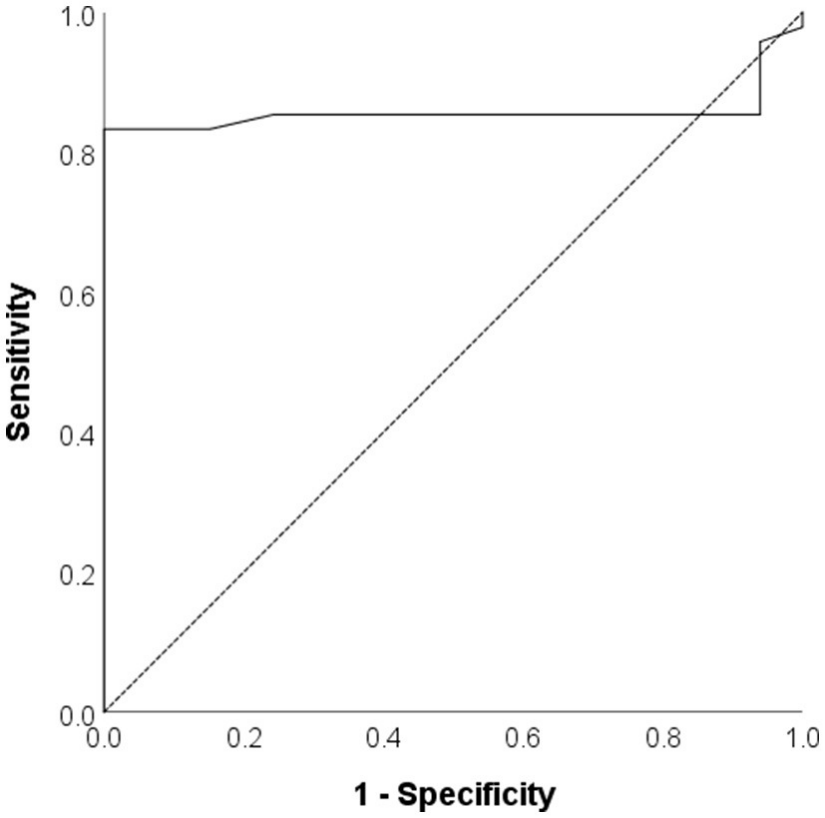
The Receiver operating curve for transverse rectal diameter in distinguishing children with upper urinary tract deterioration.

The ROC analysis revealed that transverse rectal diameter has good performance for distinguishing children with upper urinary tract deterioration, with an AUC of 0.857 (95% CI 0.761–0.953) (Figure 2).

A transverse rectal diameter value of 40 mm or greater was 83.3% sensitive and 100% specific for the diagnosis of unfavorable urodynamic patterns.

Urodynamic parameters compared according to transverse rectal diameter cutoff of ≥40 mm are presented in Table 2.

**Table 2:** Urodynamic findings of study population by transversal rectal diameter.

**Variable**	**Rectal diameter < 40 mm**	**Rectal diameter ≥ 40 mm**	***p***
Maximum bladder capacity (ml)	247 (189–298)	112.5 (83–134.5)	<0.001
MBC % EBC (ml)	330 (240–390)	330 (225–360)	<0.001
Maximal detrusor pressure (cmH20)	36 (31–39)	75 (69–83.5)	<0.001
Compliance (ml/cmH20)	6 (5.1–7.5)	2.5 (2–3.1)	<0.001
Leak point pressure (cmH20)	32 (29–39)	69 (65–70)	<0.001
PVR (ml)	78 (68–97)	171.5 (149–187)	<0.001
PVR% EBC (ml)	22.4 (19.5–26.9)	62.1 (53.9–86.7)	<0.001

Med (25–75 th percentile).

Maximum bladder capacity (MBC) expressed as % EBC and compliance were significantly lower in children with transverse rectal diameter ≥40 mm. Maximal detrusor pressure, leak point pressure, and PVR were significantly higher in children with transverse rectal diameter ≥40 mm.

## Discussion

For the longest time, it has been acknowledged that children who did not have other urological deformities could be affected by constipation through urinary tract infections, lower urinary tract function, and urinary incontinence (3, 4).

Anatomical proximity of the bladder and urethra to the rectum, alongside their symbolic embryological origin and analogous innervation, has just been one of many theories used in revealing the connection between constipation and lower urinary tract dysfunction (10).

Another possible answer could lie in the fact that when a considerable amount of fecal matter is maintained in the sigmoid colon and rectum, bladder activity is either totally blocked or its capacity and contractility are significantly impacted (where a reduction in functional capacity *via* stimulation of an increase in bladder contractility) (11) due to the compression all together with its neck (12).

In addition, a strong bond between the bladder and bowel in the central nervous system can be used as another explanation (13).

As evidenced by the data of patients with imperforate anus, contraction of the colon can also cause contraction of the bladder *via* common neural pathways in the spinal cord or pelvis (14).

In the recent past, Pezzone et al. and Ustinova et al. reported that bladder overactivity in rats is induced by acute colitis, and so provides experimental evidence of cross talk amid various pelvic viscera (15, 16).

An observation of this was recorded by Israel Franco, where an acute relapse of the disease is also commonly associated with increased micturition in patients suffering from colitis. When colitis is properly managed, urinary symptoms also discontinue. Under these circumstances, the patient may display dysuria and frequency/urgency. Moreover, the author established that in many situations, the aid of a sound bowel program was able to help in amending discomfort where boys displayed chronic erections and where younger girls complained with regard to chronic perineal or vaginal discomfort. The abovementioned cross talk mechanisms justify the data found in those patients suffering from colitis, imperforate anus, as well as regular children who encounter an immediate urge to urinate consequent to a contraction of the colon or gaseous distention, which can be undetectable to them in some cases. The significance of an adequate bowel management program with regard to the treatment of an overactive bladder syndrome is highlighted in this cross talk mechanism (17).

It follows from the above that contractions in the rectum that result in bladder overactivity through common neural pathways, navigating up the sympathetic nerves, are independent of rectal proximity to the bladder or the compression of the bladder by stool. Had compression been a relevant component, every pregnant woman would experience hyperactivity regarding the bladder with detectable urinary incontinence; however, frequency is present as opposed to urinary incontinence.

The diagnosis of constipation is unreliable if the main symptoms of the Roman III/IV criteria are not sufficient. In these situations, further X-rays, radiography, diagnostic information from a rectal exam, or ultrasound scans may be helpful. The ultrasound-measured transverse diameter regarding the rectum could be utilized as a valid additional accessory in the diagnosis of constipation when it comes to children. In addition, major factors included in persuading parents and patients of the necessity of constipation therapy are the visualization of the fecal mass and the dilated rectal impression in the bladder. Successful therapy can be followed by ultrasonography without a new rectal exam (9).

This assessment is characterized to be of vital importance when considering the diagnosis of functional constipation in children, despite the numerous controversial arguments which envelop the prognostic valuation of transverse rectal diameter in the investigation of functional constipation (6, 7).

Numerous authors have conveyed their opinions regarding how digital rectal examination has the possibility of being interchanged through the identification of transverse rectal diameter as a proper guideline while being at the leading edge when it comes to the examination of functional constipation (9, 18, 19).

In addition, a substantial discrepancy has been identified during the investigation of the cutoff point used in determining a relationship between increased rectal diameter and functional constipation, with the expected cutoff points stretching between 2.4 and 4 cm (5, 9, 20, 21).

Burgers et al. (5) did not establish a correlation between the transversal diameter and age, which is also supported by Joensson et al. (19), but it is also a fact that studies are emphasizing the existence of that correlation.

Nonetheless, based on the current research, only a few studies are focusing on the connection between bowel and bladder dysfunction in children suffering from neurogenic bladder and bowel dysfunction. Amid those limited present studies that are accessible, a specific study by Kort et al. produced zero analytically applicable alterations being recorded in urodynamic parameters after bowel management in those patients suffering from spina bifida was administered. When taking into consideration leak point pressure, bladder capacity, and instability, there was an insignificant amount of changes documented (2).

Nonetheless, research conducted by Cameron et al. that examined the extremity of bowel dysfunction in patients with neurogenic bladder showed disagreeing results. Lower urinary tract and bladder incontinence manifestations are linked straight with bowel symptoms, highlighting the importance of recognizing and treating bladder dysfunction simultaneously with urinary issues (22).

A recent study that we conducted accurately indicates how bowel management being administered might potentially be beneficial in terms of bladder function and urodynamic analysis in children suffering from detrusor sphincter overactivity and dyssynergia (23).

A reduction in maximal detrusor pressure and an increase in maximum bladder capacity and compliance serve as a confirmation of the effect. Furthermore, a substantial reduction in both PVR and leak point pressure was established in patients who were capable of accomplishing spontaneous voiding.

In children with neurogenic bowel and bladder dysfunction, we have not investigated the equivalence between the transverse rectal diameter and their urodynamic findings until now.

Therefore, the focus of this study was just to examine that particular correlation.

Our findings in this study showed that the transverse rectal diameter was substantially positive with regard to maximal detrusor and leak point pressure and PVR, as well as substantially negative with regard to maximum bladder capacity and compliance.

It has been identified that the transverse rectal diameter measured by ultrasound was undeniably a clinically adequate criterion for the recognition of children with neurogenic bowel and bladder dysfunction who were sensitive to upper urinary tract deterioration, by analyzing the diagnostic importance of transverse rectal diameter in anticipating a urodynamically undesirable prognosis.

When compared to children with a positive urodynamic prognosis, those with a negative one had a transverse rectal diameter that was considerably expanded.

Through ROC analysis, it has been established in this study that transverse rectal diameter can be qualified for having a satisfactory performance in determining children who suffer from upper urinary tract deterioration, with an AUC reading of 0.857 (95%CI 0.761–0.953).

In diagnosing the disadvantageous urodynamic prognosis, transverse rectal diameter ≥40 mm was verified to be the most desirable cutoff value.

As a result, we can confirm that there is an equivalence between the transverse rectal diameter and urodynamic findings in children suffering from neurogenic bowel and bladder dysfunction.

If there are adverse findings in the transverse diameter of the rectum, it is advisable to change the bowel/bladder treatment immediately before urodynamic studies.

However, we find it difficult to explain the exact mechanism of how the transverse rectal diameter and urodynamic parameters are associated in children with neurogenic bowel and bladder dysfunction.

Nevertheless, we will continue to search for explanations for these queries in forthcoming studies.

Determining the transverse diameter of the rectum is not intended to replace urodynamic evaluation, but it is an auxiliary diagnostic procedure that should become an integral part of the evaluation in children with neurogenic bowel and bladder dysfunction. This is extremely important while awaiting urodynamic testing while taking into consideration the degree to which urodynamic studies are capable of being problematic, costly, time-consuming, invasive, technically demanding, and of decreased availability with regard to children. For children suffering from neurogenic bowel and bladder dysfunction who may have an unfavorable urodynamic prediction, it is essential to establish an effortless, uncomplicated, and noninvasive method for their identification.

The restraints of this investigation include the absence of a control group present in children without spinal dysraphism and a limited number of studied patients. According to the fact that transverse rectal diameter was not evaluated in children without spinal dysraphism, the correlation between levels in patients at small risk and in children without spinal dysraphism was not measured. Moreover, there are no studies performed regarding patients who have neurogenic bladder as well as other findings including extensive capacity, satisfactory compliance, or areflexic bladders, of which the inclusion would be useful in establishing the practicality of transverse rectal diameter.

An important methodological limitation is that this study is not specifically designed to find a difference, so there is not a predefined difference calculated. In addition, a proper prospective analysis is needed to find significant results.

In addition, the limitations regarding the investigation involve the difficulty linked with accurately monitoring the extent of bladder filling after only ultrasonography without cystometry is executed, considering that we will determine the degree of bladder fullness according to age exclusively with the help of ultrasound.

Additional limitations of the study include the fact that the results are limited to children at the age of 9.5 ± 3.4 years, which especially refers to the cutoff of 4 cm, bearing in mind that the rectum enlarges with age, especially in chronically constipated children.

The abovementioned disadvantages must be considered before conducting any additional research.

## Conclusion

There is a correlation between the transverse rectal diameter and urodynamic findings in children with neurogenic bowel and bladder dysfunction. Ultrasonographically assessed transverse rectal diameter ≥40 mm may be used as a risk factor for upper urinary tract deterioration (unfavorable urodynamic findings). We suggest the transverse rectal diameter echosonographic measurement use as an integral part of the diagnostic approach in children with neurogenic bowel and bladder dysfunction, as it can help decision-making while waiting for urodynamic testing.

## Data availability statement

The datasets used and analyzed during the current study are available from the corresponding author on reasonable request.

## Author contributions

SM: conceptualization and design of the study, drafting of the manuscript, critical review of the manuscript, and approval of the final manuscript. JM-L: conceptualization and design of the study, data analysis and interpretation, drafting of the manuscript, and approval of the final manuscript. OR, DL, AZ, ID, and ZR: conceptualization and design of the study, drafting of the manuscript, and approval of the final manuscript. NM: conceptualization and design of the study, data analysis and interpretation, critical review of the manuscript, and approval of the final manuscript. All authors contributed to the article and approved the submitted version.

## Conflict of interest

The authors declare that the research was conducted in the absence of any commercial or financial relationships that could be construed as a potential conflict of interest.

## Publisher's note

All claims expressed in this article are solely those of the authors and do not necessarily represent those of their affiliated organizations, or those of the publisher, the editors and the reviewers. Any product that may be evaluated in this article, or claim that may be made by its manufacturer, is not guaranteed or endorsed by the publisher.
